# Atypical functional connectivity of temporal cortex with precuneus and visual regions may be an early-age signature of ASD

**DOI:** 10.1186/s13229-023-00543-8

**Published:** 2023-03-10

**Authors:** Yaqiong Xiao, Teresa H. Wen, Lauren Kupis, Lisa T. Eyler, Vani Taluja, Jaden Troxel, Disha Goel, Michael V. Lombardo, Karen Pierce, Eric Courchesne

**Affiliations:** 1 Center for Language and Brain, Shenzhen Institute of Neuroscience, Shenzhen, 518107 China; 2 Autism Center of Excellence, Department of Neurosciences, University of California, San Diego, La Jolla, CA 92037 USA; 3 Department of Psychology, University of Miami, Coral Gables, FL USA; 4 Department of Psychiatry, University of California, 9500 Gilman Drive, La Jolla, San Diego, CA 92161 USA; 5 VISN 22 Mental Illness Research, Education, and Clinical Center, VA San Diego Healthcare System, San Diego, CA USA; 6 Laboratory for Autism and Neurodevelopmental Disorders, Center for Neuroscience and Cognitive Systems UniTn, Istituto Italiano di Tecnologia, Rovereto, Italy

**Keywords:** Autism spectrum disorder, Superior temporal cortex, Resting-state functional connectivity, Language networks, Social visual attention

## Abstract

**Background:**

Social and language abilities are closely intertwined during early typical development. In autism spectrum disorder (ASD), however, deficits in social and language development are early-age core symptoms. We previously reported that superior temporal cortex, a well-established social and language region, shows reduced activation to social affective speech in ASD toddlers; however, the atypical cortical connectivity that accompanies this deviance remains unknown.

**Methods:**

We collected clinical, eye tracking, and resting-state fMRI data from 86 ASD and non-ASD subjects (mean age 2.3 ± 0.7 years). Functional connectivity of left and right superior temporal regions with other cortical regions and correlations between this connectivity and each child’s social and language abilities were examined.

**Results:**

While there was no group difference in functional connectivity, the connectivity between superior temporal cortex and frontal and parietal regions was significantly correlated with language, communication, and social abilities in non-ASD subjects, but these effects were absent in ASD subjects. Instead, ASD subjects, regardless of different social or nonsocial visual preferences, showed atypical correlations between temporal–visual region connectivity and communication ability (*r*(49) = 0.55, *p* < 0.001) and between temporal–precuneus connectivity and expressive language ability (*r*(49) = 0.58, *p* < 0.001).

**Limitations:**

The distinct connectivity–behavior correlation patterns may be related to different developmental stages in ASD and non-ASD subjects. The use of a prior 2-year-old template for spatial normalization may not be optimal for a few subjects beyond this age range.

**Conclusions:**

Superior temporal cortex is known to have reduced activation to social affective speech in ASD at early ages, and here we find in ASD toddlers that it also has atypical connectivity with visual and precuneus cortices that is correlated with communication and language ability, a pattern not seen in non-ASD toddlers. This atypicality may be an early-age signature of ASD that also explains why the disorder has deviant early language and social development. Given that these atypical connectivity patterns are also present in older individuals with ASD, we conclude these atypical connectivity patterns persist across age and may explain why successful interventions targeting language and social skills at all ages in ASD are so difficult to achieve.

**Supplementary Information:**

The online version contains supplementary material available at 10.1186/s13229-023-00543-8.

## Background

Early social and communication deficits are often early warning signs of autism [1, 2]. Research with typically developing (TD) infants and toddlers has led to a theory that language and social development are closely intertwined in the early years of life [3, 4]. Specifically, social attention and social interactions are crucial for early language learning [5–8]. It is well recognized that typical language development is contingent on and constrained by early reciprocal social engagement [6, 9], and that early language learning is associated with the emergence of the social brain in infants [3, 6]. A prominent theory is that the brain substrate for this is superior temporal cortex, including both superior temporal gyrus (STG) and sulcus (STS), because this region is critical to processing language, auditory, and visual social inputs [10–14]. The connectivity of this region with prefrontal, cingulate and parietal cortical areas is critical both for the normal developmental acquisition of social and speech perception and for learning the communicative significance of these inputs [11]. In individuals with autism spectrum disorder (ASD), impairments in superior temporal cortex function are therefore theorized to underlie social and language abnormalities seen in the disorder [11, 15]. Studies have reported that autistic children’s impaired behavioral response to social affective speech (such as “motherese” speech) may be linked to neural responses to speech [16] and that neural responses to known words in children with ASD with less severe social symptoms predict language outcome at ages 4 and 6 [17]. In another early study, ASD toddlers exhibited significantly weaker inter-hemispheric correlation in two key language areas, superior temporal cortex and inferior frontal gyrus (IFG), and the lower IFG inter-hemispheric connectivity was associated with reduced language ability and worse ASD social and communication symptoms [18]. Despite these important early studies, only more recently has there been research in toddlers confirmed as ASD that has examined whether and how functional connectivity dysregulation in language and social neural networks relates to language and social behavioral symptoms in the early development of ASD.

Recent fMRI activation studies have robustly and reproducibly demonstrated that superior temporal cortex (including both STG and STS) show reduced activation to speech in ASD toddlers [15, 19–22]. ASD toddlers with the least neural response to social affective speech have the lowest language and social abilities [20, 22]. Toddlers with ASD with relatively high symptom severity are also more likely to exhibit early brain overgrowth and atypical gene expression as compared to toddlers with ASD with relatively low symptom severity and typical toddlers [21]. Moreover, those toddlers with ASD with the most extreme reduction of superior temporal activation to motherese speech also exhibited the least behavioral preference for and attention towards females speaking motherese in eye tracking paradigms. This lack of neural and behavioral responsiveness is accompanied by the most severe ASD symptoms and poor language and social outcomes [22]. We infer that these extreme neural-behavioral deficits may explain social attention deficits, which are distinctive in ASD.

The superior temporal cortex, a core region supporting language and social processing, is interconnected with additional downstream regions including inferior frontal cortex, anterior cingulate cortex (ACC), dorsolateral prefrontal cortex (DLPFC), lateral parietal cortex (LPC), and posterior cingulate cortex/precuneus (PCC) that also underlie language, social affect, communication, and speech functions [11, 13, 23–26]. Therefore, in ASD toddlers, reduced neural responsiveness to social language stimuli in superior temporal cortex may have widespread negative effects on language, speech, and social learning, and deviant connectivity may be correlated with abilities in these social and language domains in affected toddlers.

Previous studies have consistently reported atypical activation in visual (cuneus) cortex or connectivity with the visual (cuneus) cortex in older children, adolescents, and adult with ASD [27–31]. These findings raise the possibility of abnormal involvement of visual cortex in social and language processing in ASD infants and toddlers as well. In fact, abnormal social visual attention has been identified as a significant feature for different ASD subtypes (e.g., social vs. non-social responders) by several eye tracking studies with large samples (*N* = 100, 334, and 1,863 toddlers) [32–34], but whether this subtype might drive aberrant connectivity of superior temporal cortex with visual regions at the age of first diagnosis in ASD toddlers has never been investigated. In a recent study, connectivity of superior temporal cortex with PCC—a core region of the default mode network—was reported, and a subgroup of ASD children showed a mediation effect of PCC on connectivity between language and visual regions [28]. These findings in older individuals with ASD are indicative of abnormal involvement of visual regions and PCC among ASD subjects or an ASD subgroup, which may be biologically meaningful and behaviorally relevant. However, temporal cortical connectivity with downstream or aberrant upstream systems and the correlations between this connectivity and social and language ability in confirmed toddlers and young children with ASD, have never been studied and remain unknown.

In the only tractography study of its kind [35], we found evidence of an excess of abnormally small weakly maturing axons in multiple language and social fiber tracts, including the arcuate fasciculus and superior longitudinal fasciculus that connect posterior superior temporal and supramarginal and angular regions with lateral prefrontal cortex. Combined with reduced social and language activation, this axonal pathobiology predicts diminished functional effectiveness of connectivity between temporal, frontal, and parietal regions in supporting language and social development in ASD.

Here we examined the foundational question of whether there is aberrant functional connectivity of the superior temporal cortex (a region involved in both language and social functions) and how superior temporal cortex connectivity correlates with behavior in children with ASD at very early ages. To do so, we examined superior temporal cortex functional connectivity and tested correlations between connectivity patterns and language and social communication abilities in a large sample of toddlers with and without ASD (mean age 2.3 ± 0.7 years). We focused on left and right superior temporal regions of interest, which were those that reproducibly and robustly displayed reduced neural responsiveness to speech stimuli in multiple separate, independent cohorts of ASD toddlers [15, 19–22]. We predicted that there would be correlations between connectivity of superior temporal region and language and social regions and language and social abilities in toddlers without ASD, while such connectivity would be weaker, absent, or atypical in ASD toddlers. We also sought to determine whether aberrant connectivity in ASD might be present in an ASD subgroup with reduced social visual attention as indexed by eye tracking data, or across all ASD subjects regardless of social visual attention preference.

## Materials and methods

### Participants

This study was approved by the University of California, San Diego Institutional Review Board. Informed consent was obtained from parents or guardians of participants.

Applying an identical approach used in previous reports [20, 32, 36–39], we recruited a new cohort of toddlers through community referral and a population-based screening method in collaboration with pediatricians called the *Get SET Early* [40], which was formerly known as the 1-Year Well-Baby Check-Up Approach [38, 39]. Eighty-six young children from this new cohort participated in the present study. Their mean age was 2.3 ± 0.7 years, with 85 of the 86 subjects aged 1–3 years and 1 aged 4 years at the clinical testing. At the time of MRI scan collection, 84 of the 86 were 1–3 years old and 2 were 4 years old. Participants were assessed using the Autism Diagnostic Observation Schedule (ADOS-2; Module T, 1, or 2) [41], Mullen Scales of Early Learning [42], and Vineland Adaptive Behavior Scales (Second Edition) [43]. The Mullen assesses cognitive ability and development at ages 0 to 68 months; the Vineland assess adaptive communication, social, and behavioral functions. A diagnosis of ASD was given based on the DSM-5 diagnostic classification. Participants with initial diagnostic and clinical evaluations at < 36 months returned for follow-up evaluations. Clinical scores from a child’s most recent evaluation were used as the best estimate of abilities (for demographic information and clinical scores, see Table 1). ﻿Assessments were administered by licensed, Ph.D.-level clinical psychologists and occurred at the University of California, San Diego Autism Center of Excellence. The clinical, behavioral, and resting-state fMRI data were collected between 2018 and 2020; the resting-state fMRI data have not been published elsewhere.

**Table 1:** Demographic information and clinical test scores for ASD and non-ASD subjects

Characteristics at fMRI scan or outcome	ASD (*N* = 51) Mean(SD)	Non-ASD (*N* =35) Mean(SD)	*p* value	Cohen’*s d*
*Demographics*				
Sample size (M/F)	41/10	21/14	0.068^a^	﻿—
Age at fMRI scan, months	28.55 (9.2)	25.89 (8.51)	0.172	0.3
Age at clinical tests, months	27.51 (8.62)	26.34 (8.21)	0.64	0.14
Eye tracking				
Sample size (M/F)	25/7﻿	21/12	0.31^a^	
Age at eye tracking, months	22.38 (7.59)	19.29 (5.76)	0.07	0.46
*ADOS (module T, 1, or 2) score*				
ADOS SA	13.88 (3.62)	2.97 (1.64)	< 0.001	3.66
ADOS RRB	5.55 (1.93)	1.54 (1.5)	< 0.001	2.26
ADOS Total	19.43(4.77)	4.51 (2.19)	< 0.001	3.79
*Mullen T score*				
Visual reception	36.55 (13.53)	53.91 (11.86)	< 0.001	1.35
Fine motor	38.16 (12.67)	49.86 (8.27)	< 0.001	1.1
Receptive language	30.35 (14.59)	46.66 (11.75)	< 0.001	1.21
Expressive language	30.18 (16.55)	43.11 (12.48)	< 0.001	0.86
Early learning composite	71.78 (20.37)	97.09 (16.6)	< 0.001	1.34
Mullen age equivalent adjusted language score^*^				
Receptive language	59.92 (30.86)	96.12 (21.13)	< 0.001	1.32
Expressive language	62.01 (28.72)	87.13 (22.95)	< 0.001	-0.95
*Vineland standard score*				
Communication	81.55 (16.8)	96.26 (10.67)	< 0.001	1.01
Daily living skills	86.41 (11.81)	96.29 (10.83)	< 0.001	0.87
Socialization	82.86 (12.14)	96.6 (9.82)	< 0.001	1.22
Motor skills	91.58 (10.33)	98.41 (9.53)	0.003	0.68
Adaptive behavior composite	81.88 (11.33)	94.94 (9.72)	< 0.001	1.22

*ASD* Autism spectrum disorder, *ADOS* Autism Diagnostic Observation Schedule, *SA* Social affect, *RRB* Restricted and repetitive behavior

^a^ Pearson’s chi-squared test (otherwise, the *p* values are from Welch’s *t* tests)

^*^ Mullen age equivalent adjusted language score was calculated: (age equivalent scores/age in months)*100%

Among 86 subjects (51 ASD, 35 non-ASD), 31% of ASD and 40% of non-ASD subjects were from families reporting over $100,000 in total family income; 43% of ASD and 49% of non-ASD subjects from families reporting family income between $20,000–$100,000. For parental education, one or both parents of 63% of ASD subjects and 63% of non-ASD subjects had a college, masters, or professional degree. Regarding language exposure at home, 61% of ASD and 54% of non-ASD subjects were from multilingual families; 71% of ASD and 77% of non-ASD subjects were from families where English was used always, and for the remainder English was spoken sometimes.

Resting-state fMRI data were collected from all 86 subjects (51 ASD, 35 non-ASD) during natural sleep. Participants were considered non-ASD if their diagnosis at the outcome visit was non-ASD and their Mullen Early Learning Composite score fell within 2 standard deviations of the mean score (i.e., > 70). This allowed for examination of brain functional connectivity along a continuum of language and cognitive abilities from below to above average in non-ASD children.

### GeoPref eye tracking test

All 86 subjects also participated in the GeoPref eye tracking test, which indexes social visual attention [32, 34, 36, 38]. In the GeoPref Test, subjects watched two silent movies displaying dynamic geometric or dynamic social stimuli side by side. Dynamic geometric stimuli consisted of colourful moving geometric patterns and dynamic social images consisted of children doing yoga exercises. Quality GeoPref scores were obtained from 32 subjects with ASD and 33 with non-ASD. ASD subjects were further divided into nonsocial visual responders (ASD_nonSoc_) and social visual responders (ASD_Soc_) based on a threshold of 69% fixation to dynamic social stimuli [33] (see Additional file 1: Methods for *GeoPref eye tracking test*).

### MRI data acquisition

Structural and functional MRI data were collected in a 3 T GE scanner at the University of California, San Diego Center for Functional MRI. Resting-state functional images were acquired with a multi-echo EPI protocol (TE = 15 ms, 28 ms, 42 ms, 56 ms; TR = 2500 ms; flip angle = 78°; matrix size = 64 × 64; slice thickness = 4 mm; field of view (FOV) = 256 mm; 34 slices, 288 volumes, a total of 12 min). ﻿Structural images were ﻿acquired using a T1-weighted MPRAGE sequence (FOV = 256 mm; TE = 3.172 ms; TR = 8.142 ms; Flip angle = 12°).

### Imaging data preprocessing

Multi-echo resting-state fMRI data were preprocessed using Multi-Echo Independent Components Analysis (ME-ICA) with a pipeline “meica.py” (ME-ICA 3.2) [44, 45] implemented in AFNI [46] and Python. Prior to preprocessing, the first 4 volumes were discarded to allow for magnetization to reach steady state. Preprocessing before data denoising included motion correction, slice timing correction for images of each TE, spatial normalization using an age-matched toddler template (i.e., 2-year-old template) [47] as the majority of the subjects fell into this age range (mean age: 2.3 ± 0.7 years), and optimal combination of time series of all TEs. The head motion correction was conducted using the rigid-body alignment (AFNI’s 3dvolreg function) based on the first TE images (TE = 15 ms), which yielded 6 parameters as quantification of head motion. For spatial normalization, fMRI volumes were first registered to native subject’s T1 space applying a linear registration using AFNI’s 3dAllineate function and then registered to the template space using AFNI’s 3dQWarp function applying a non-linear affine registration. For detailed description of ME-ICA process, see Additional file 1: Methods for *ME-ICA procedure*. The final multi-echo denoised images (voxel size: 3.35 mm × 3.35 mm × 3.35 mm) were used for subsequent seed-based connectivity analysis.

We further quantified head motion via framewise displacement (FD) and no group differences were observed (see Additional file 1: Methods for *Group comparisons of head motion*).

### Seed-based functional connectivity analysis

We selected two language-relevant regions of interest (ROIs), i.e., left and right superior temporal regions, from the meta-analytic activation map in Neurosynth (https://neurosynth.org/) with the term “language”. These ROIs were identical to those used in our previous studies [20, 21] and are displayed in Additional file1: Figure S1. As these ROIs are from adult samples, they were co-registered to the age-matched template using FSL’s flirt function [48, 49].

For estimating seed-based functional connectivity, we used a multiple-echo independent component regression (ME-ICR) approach, which has been shown to be superior at estimating seed-based connectivity while adjusting for effective degrees of freedom based on the BOLD-dimensionality of each individual’s data [45, 50]. Pearson’s correlation coefficients were computed on the “mefc” images, the ICs output from the ME-ICA pipeline. Computing seed-based connectivity based on this data has been shown to be a robust estimator of functional connectivity and allows for appropriate adjustment for effective degrees of freedom, denoted by the number of ICs, which vary from subject to subject [45]. Fisher *Z*-transformation were computed on the connectivity maps, and then a 6 mm FWHM smoothing kernel was applied to enhance SNR before group-level comparisons.

### Connectivity–behavior correlation analysis

For the main analysis, we investigated relationships between iFC of language ROIs and language, communication, and social scores as assessed by the Mullen (Mullen expressive and receptive subscales) and Vineland (Vineland communication and socialization subscales) (see Additional file 1: Methods for *Characteristics of Mullen and Vineland subtests*). Specifically, we implemented the group analyses for ASD and non-ASD subjects via the 3dMVM program [51] in AFNI [46] using the following formula:

$$\begin{aligned} iFC =\; & \beta_{0} + \beta_{1} \times {\text{group}} + \beta_{2} \times {\text{ behavioral measure }} + \beta_{3} \times {\text{ group}} \times {\text{behavioral measure}} + \beta_{4} \times {\text{ age}} \\ & + \beta_{5} \times {\text{gender }} + \beta_{6} \times {\text{mean }}FD + \varepsilon \\ \end{aligned}$$

In the regression model, group, behavioral measure, and their interactions were covariates of interest, while age, gender, and mean FD were covariates of no interest. The behavioral measures included Mullen age equivalent adjusted expressive and receptive language scores (see Additional file 1: Methods *for Calculation of Mullen age equivalent adjusted scores*) and Vineland communication and socialization scores, which index a child’s language, communication, and social ability [42, 43]. Among the output of the regression analysis, we focused on: (1) main effects of group and behavioral measure, (2) interaction effects of group and connectivity–behavior correlations, and (3) connectivity–behavior correlations within each group. Resulting correlation coefficient maps were transformed to *t*-maps and corrected for multiple comparisons with the family-wise error (FWE) approach using 3dClustSim program in AFNI with a threshold of cluster-wise *p* < 0.05 (at voxel-wise *p* = 0.001 as suggested by a previous study [52] and cluster size > 63 voxels). This spatial cluster correction took into account spatial autocorrelation by using the ‘–acf’ option in 3dClustSim.

### Analysis in ASD eye tracking subtypes

To test the a priori hypothesis of different temporal–visual cortex connectivity relationships with social and language ability in ASD eye tracking subgroups, we first extracted *Z*-transformed correlation coefficients from clusters that exhibited significant correlations between superior temporal cortex connectivity and behavioral measures in ASD subjects. Next, we examined the correlations in ASD_Soc_ and ASD_nonSoc_ subgroups separately and presented scatterplots with trend lines for each group. ﻿Considering the relatively small sample sizes in the two ASD subgroups, to estimate correlation coefficients, we performed bootstrapping for the correlations in each ASD subgroup (ASD_Soc_ and ASD_nonSoc_) separately using the ‘boot’ function in the R ‘boot’ package to compute 95% confidence intervals (CI) around sample correlation estimates (100,000 bootstrap resamples). This analysis allows for reporting the distribution of sample correlation estimates that could have been observed. Finally, we tested correlation strength differences between ASD_Soc_ and ASD_nonSoc_ subgroups using the paired.r function in R ‘psych’ package.

## Results

### Non-significant main effects of group or behavioral measure

We did not observe main effects of group or behavioral measure after correcting for multiple comparisons at voxel-wise *p* = 0.001 and cluster size > 63 voxels (cluster-wise *p* < 0.05; FWE corrected).

### Interaction effects of group and connectivity–behavior correlations

**Fig. 1:**
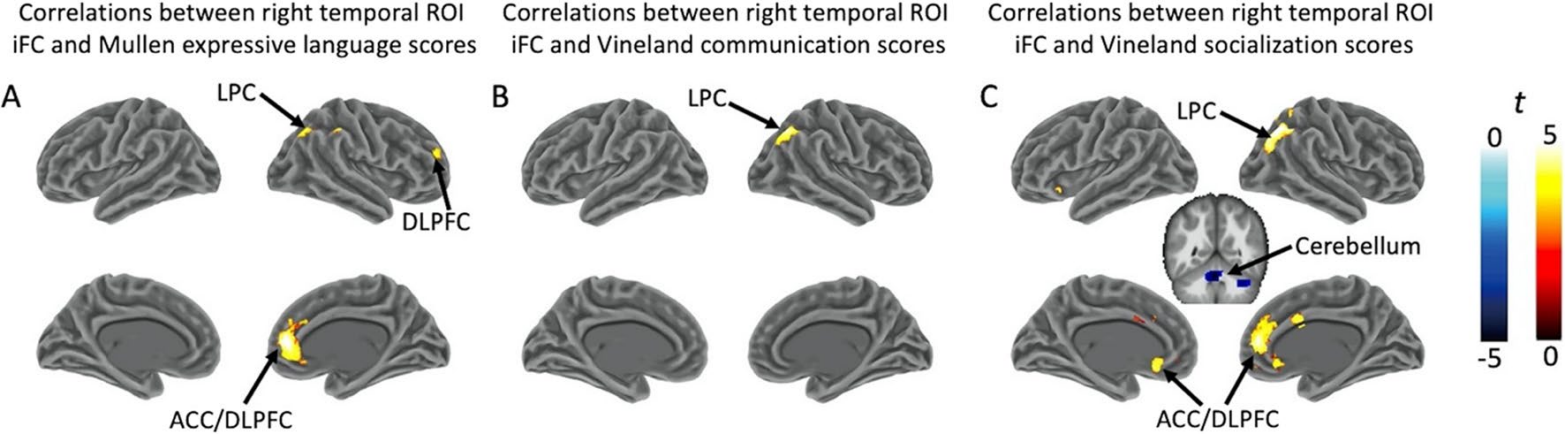
Clusters showing significant interactions between group and connectivity–behavior correlations. These interaction effects included: **A** right ACC/DLPFC and right LPC for correlations between iFC of the right temporal ROI and Mullen age equivalent adjusted expressive language scores; **B** right LPC for correlations between iFC of the right temporal ROI and Vineland communication scores; **C** bilateral ACC/ DLPFC, right LPC, and right cerebellum for correlations between iFC of the right temporal ROI and Vineland socialization scores. Clusters were corrected for multiple comparisons with voxel-wise *p* = 0.001 and cluster size > 63 voxels (cluster-wise *p* < 0.05, FWE corrected). *ROI* Region of interest, *iFC* Intrinsic functional connectivity, *LPC* Lateral parietal cortex, *ACC* Anterior cingulate cortex, *DLPFC* Dorsolateral prefrontal cortex

We observed significant interaction effects of group and connectivity–behavior correlation (Fig. 1 and Additional file 1: Table S1). Specifically, for correlations between iFC of the right temporal ROI and Mullen age equivalent adjusted expressive language scores, interaction effects were found in the right ACC/DLPFC and the right LPC (Fig. 1A). The interaction effects were also found in the right LPC for correlations between iFC of right temporal ROI and Vineland communication scores (Fig. 1B), and in the bilateral ACC/DLPFC, right LPC, and right cerebellum for correlations between iFC of the right temporal ROI and Vineland socialization scores (Fig. 1C).

### Significant connectivity–behavior correlations in non-ASD and ASD subjects

**Fig. 2:**
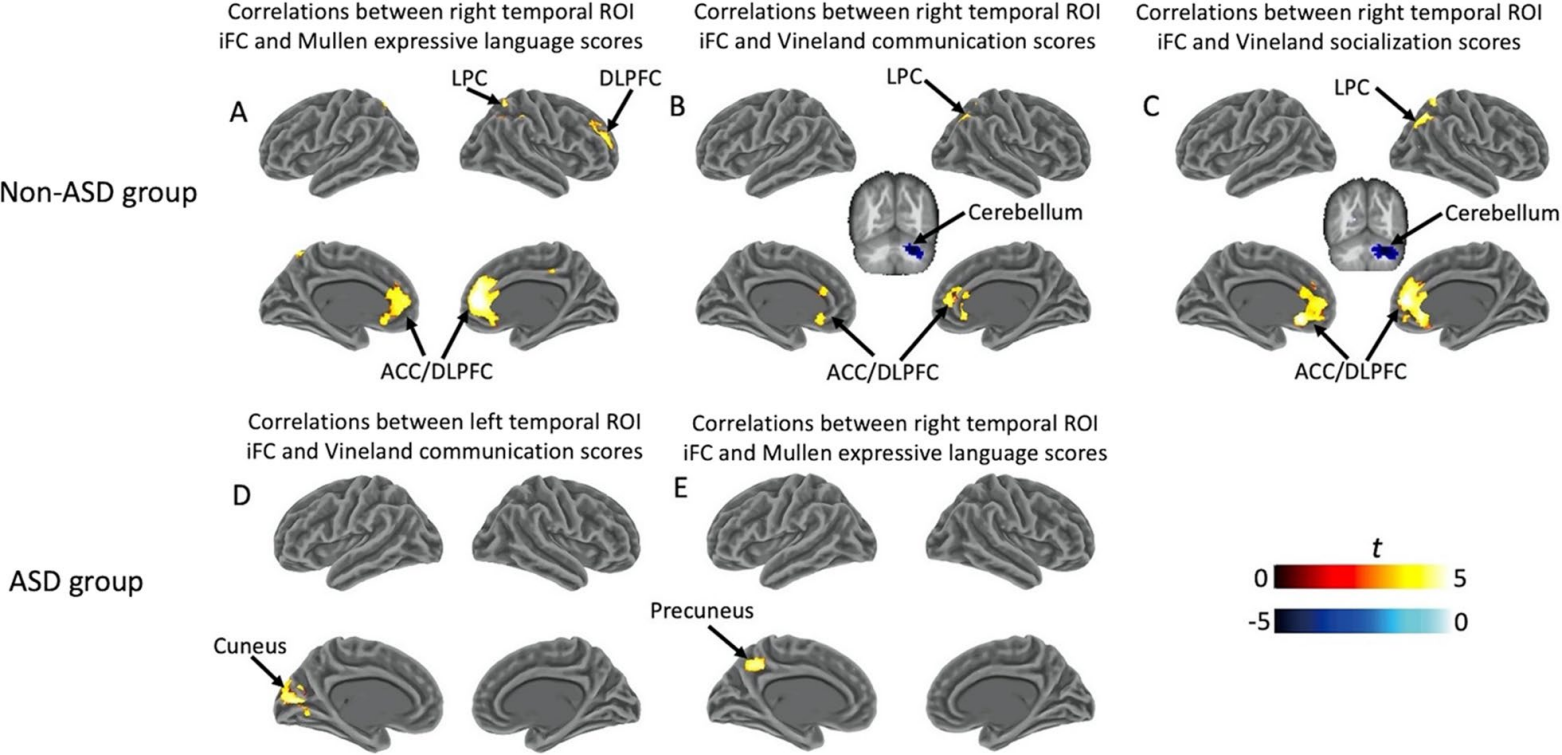
Clusters showing significant connectivity–behavior correlations in non-ASD and ASD groups. In the non-ASD group, **A** iFC of right temporal ROI and both bilateral ACC/DLPFC and right LPC was correlated with Mullen age equivalent adjusted expressive language scores; **B** iFC of right temporal ROI and bilateral ACC/DMPFC, right LPC and right cerebellum linked to both Vineland communication scores; and **C** iFC of right temporal ROI and Vineland socialization scores. In the ASD group, iFC of left temporal ROI and left cuneus was correlated with Vineland communication scores; and **E** iFC of right temporal ROI and left precuneus was correlated with Mullen age equivalent adjusted expressive language scores. Clusters were corrected for multiple comparisons (cluster-wise *p* < 0.05, FWE corrected). *iFC* Intrinsic functional connectivity, *ACC* Anterior cingulate cortex, *DLPFC* Dorsolateral prefrontal cortex, *LPC* Lateral parietal cortex

Non-ASD subjects had multiple significant positive correlations between right temporal ROI iFC and language and social scores after multiple comparisons correction (voxel-wise *p* = 0.001, cluster size > 63 voxels, cluster-wise *p* < 0.05, FWE corrected) (Fig. 2 and Additional file 1: Table S1). These included significant right temporal ROI connectivity with bilateral ACC/DLPFC (*r*(33) = 0.61, *p* < 0.001, 698 voxels) and right LPC (*r*(33) = 0.5, *p* = 0.002, 111 voxels) that correlated with Mullen age equivalent adjusted expressive language scores (Fig. 2A). Connectivity between right temporal ROI and various brain regions also correlated with Vineland communication (Fig. 2B) and socialization scores (Fig. 2C), including bilateral ACC/DLPFC (Vineland communication: *r*(33) = 0.55, *p* < 0.001, 145 voxels; Vineland socialization: *r*(33) = 0.59, *p* < 0.001, 584 voxels), right LPC (Vineland communication: *r*(33) = 0.5, *p* = 0.002, 79 voxels; Vineland socialization:* r*(33) = 0.6, *p* < 0.001, 234 voxels), and right cerebellum (Vineland communication: *r*(33) = − 0.54, *p* < 0.001, 113 voxels; Vineland socialization:* r*(33) = − 0.57, *p* < 0.001, 225 voxels).

These significant connectivity-behavior correlations in non-ASD subjects were not present in ASD subjects even without stringent FWE corrections. Instead, we found iFC between left temporal ROI and left cuneus significantly correlated with Vineland communication scores (*r*(49) = 0.55; *p* < 0.001, 155 voxels; Fig. 2D), and iFC between right temporal ROI and left precuneus significantly correlated with Mullen age equivalent adjusted expressive language scores (*r*(49) = 0.58; *p* < 0.001, 78 voxels; Fig. 2E).

### ASD subgroups as indexed by eye tracking performance and analyses of subgroup-specific relationships

**Fig. 3:**
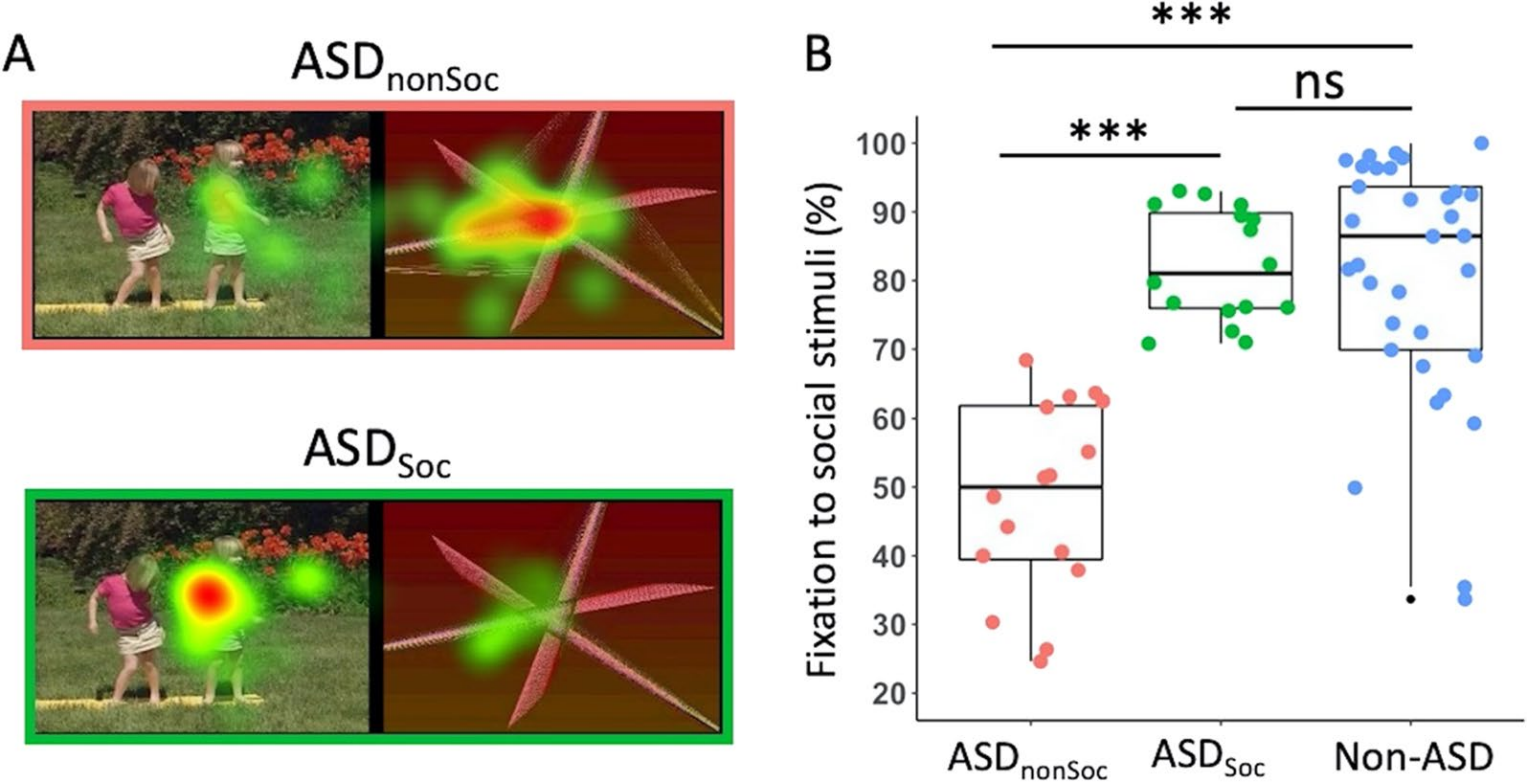
Identification of nonsocial and social visual ASD subgroups. **A** Examples of the stimuli used in the GeoPref eye tracking test; example fixation from a nonsocial visual ASD (ASD_nonSoc_) individual (pink) and a social visual ASD (ASD_Soc_) individual (green). **B** Scatter-boxplot of GeoPref test performance for subjects who also had resting-state fMRI data (ASD_nonSoc_: *n* = 16, pink; ASD_Soc_: *n* = 16, green; non-ASD: *n* = 33, blue). *** *p* < 0.001; ns Not significant

Among the 32 ASD subjects with quality eye tracking data, 16 were designated as nonsocial visual responders (i.e., ASD_nonSoc_) and 16 as social visual responders (i.e., ASD_Soc_) based on a threshold of 69% fixation to dynamic social stimuli (Figs. 3A and B). Specifically, ASD subjects were categorized as ASD_nonSoc_ subgroup if they spent < 69% of the duration of the video looking at social images, and ASD subjects were categorized as ASD_Soc_ subgroup if they spent ≥ 69% of the duration of the video looking at social images.

Next, we performed analyses to test whether aberrant temporal–cuneus iFC and temporal–precuneus iFC observed in ASD subjects were driven by the ASD_Soc_ subgroup, ASD_nonSoc_ subgroup, or both. To test this, functional connectivity values (*t* values) were extracted from the cuneus that showed significant correlations between iFC of the left temporal ROI and Vineland communication scores and from precuneus that showed significant correlations between iFC of the right temporal ROI and Mullen age equivalent adjusted expressive language scores. In both ASD_nonSoc_ and ASD_Soc_ subgroups, iFC between left temporal ROI and visual cortex (cuneus) was positively correlated with Vineland communication scores (ASD_nonSoc_: *r*(14) = 0.55, *p* = 0.03, 95% CI = [0.095, 0.84]; ASD_Soc_:* r*(14) = 0.59, *p* = 0.017, 95% CI = [− 0.22, 0.9]), and iFC between right temporal ROI and precuneus was positively correlated with Mullen age equivalent adjusted expressive language scores (ASD_nonSoc_:* r*(14) = 0.74, *p* = 0.001, 95% CI = [0.4, 0.91]; ASD_Soc_:* r*(14) = 0.62, *p* = 0.01, 95% CI = [0.32, 0.83]). There were no significant differences between ASD_Soc_ and ASD_nonSoc_ subgroups in strength of correlation between left temporal–visual cortex connectivity and Vineland communication scores (*z* = 0.15, *p* = 0.88, two-tailed) and between right temporal–precuneus connectivity and Mullen age equivalent adjusted expressive language scores (*z* = 0.54, *p* = 0.59, two-tailed).

## Discussion

In non-ASD toddlers, greater connectivity between superior temporal cortex (including STG and STS) and regions supporting language and social processing (e.g., prefrontal, lateral parietal, and anterior cingulate cortices) was correlated with better language, communication, and socialization scores, whereas ASD toddlers failed to demonstrate even trends towards positive correlations. Instead, ASD toddlers showed that communication abilities correlated with temporal–visual cortex connectivity and that expressive language abilities correlated with temporal–precuneus connectivity. These differences in connectivity–behavior correlations cannot be explained by different patterns of connectivity in ASD vs. non-ASD toddlers because no main effect of group was observed. Instead, the differences reflect distinct associations of functional connectivity with language and communication skills in ASD and non-ASD groups. Thus, at early ages when language and social skills are being acquired and rapidly developing, connectivity between superior temporal cortex and both visual cortex and precuneus appears to play an atypical role in social and language development in ASD.

The atypical connectivity between left temporal cortex and cuneus in toddlers with ASD in the present study has been reported in studies of older individuals with ASD [27–31]. Research has also found increased functional connectivity between language regions and both visual cortex and precuneus in older children and adolescents with ASD as compared to typically developing subjects [28]. These findings indicate that the abnormal connectivity of superior temporal cortex with visual cortex and precuneus that was associated with language and communication processing is present from very early ages and persists throughout development. This connectivity–behavior deviance in ASD may help explain why interventions targeting social and language skills are so challenging for individuals with the disorder even in toddlerhood. Moreover, these early-age atypical connectivity patterns are also found in older individuals with ASD [27, 28, 30, 53]. Therefore, these abnormal connectivity patterns may not only underpin how language is first acquired and processed in toddlers with ASD across a range of language and communication ability levels, but may also persist across development to adulthood, which further helps explain why treatment of ASD core symptoms is such a challenge at all ages.

Previous studies in typical toddlers have consistently reported that language acquisition and development are closely linked to social experience (e.g., exposure to ﻿motherese, social responding, social interactions) in the first years of life [3, 7, 8, 54–56]. Here, our data demonstrate neural correlates (i.e., regions within language and social networks) of this in non-ASD children who represent a range of typical development from below to above average language and social scores. Specifically, in non-ASD children, connectivity between the right temporal ROI and social regions (i.e., ACC/DLPFC and LPC) linked to language and social communication abilities. This finding provides neural evidence compatible with the prevailing view that language and social development are normally closely intertwined in the early years of life [3, 4]. It further suggests the hypothesis that social experience may promote language acquisition and learning and enhance functional connectivity between language and social brain regions as a result of the history of coactivation among these regions in non-ASD children. The lack of such correlations between connectivity of language- and social-related regions and language and social ability at early ages in ASD may reflect reduced social visual attention and social experience as a result of inherently atypical brain functional connectivity.

We found that correlations between temporal–visual (cuneus) connectivity and Vineland communication scores and between temporal–precuneus connectivity and Mullen expressive language scores were present in both ASD_Soc_ and ASD_nonSoc_ subgroups, although these subgroups had different levels of social visual attention. These preliminary findings are intriguing. ASD subgroups did not differ in these correlations, indicating abnormal neural correlates of language deficits across all ASD subjects in the present study. Thus, abnormal temporal–cuneus and temporal-precuneus connectivity may be a more general signature of ASD as reported in previous research with older children [27, 28]. Nevertheless, these exploratory findings are preliminary given the small sample sizes, and thus it is important to confirm these comparisons of ASD_Soc_ and ASD_nonSoc_ subgroups in future studies with larger subgroup sample sizes.

One important finding is that our data show strong brain connectivity–behavior correlation patterns in non-ASD subjects for the right but not left temporal ROI and for language and social abilities assessed by Mullen and Vineland subtests. These findings support the view that the right superior temporal cortex is engaged in emotionally and socially relevant features of communication and expressive language at early ages in the typically developing brain. Further, the correlation patterns for language and social subtests reflect the primary abilities that each subtest taps. For example, connectivity between right temporal ROI and social regions (i.e., bilateral ACC/DLPFC, LPC) is only linked to Mullen expressive language but not Mullen receptive language scores. These findings suggest that resting-state functional connectivity analysis is a sensitive approach for detecting neural correlation patterns associated with different cognitive functions in early typical development [57].

### Limitations

The present study has two potential limitations. First, a 2-year-old template was selected for the spatial normalization of our 86 subjects whose mean age was 2.3 ± 0.7 years. While 84 of the sample (98%) were 1–3 year-olds, two subjects were at the age of 4 at the MRI scan. The fMRI results presented here may benefit from using a group-specific template to minimalize the individual brain tissue differences. Second, the different patterns of brain connectivity–behavior correlations in non-ASD and ASD may be related to lower cognitive levels in ASD than non-ASD subjects. More specifically, children with ASD may not have reached the developmental stage where particular skills are required, which may account for the brain connectivity–behavior relationships observed in the present study. However, our prior research comparing ASD toddlers with mental-age-matched non-ASD subjects indicated that ASD had reduced fMRI activation in an extended network of language-relevant regions [15]. Nonetheless, future research could include mental-age-matched non-ASD to further examine relationships between connectivity and correlations with clinical measures.

## Conclusions

In sum, the present study revealed that the strength of functional connectivity between superior temporal cortex and other social and language regions in frontal and parietal cortices are correlated with language, communication, and social abilities in non-ASD toddlers but not in ASD at early ages. ASD toddlers instead demonstrated abnormal and highly unusual connectivity patterns, including temporal–cuneus (visual) connectivity that correlated with communication ability and temporal–precuneus connectivity that correlated with expressive language ability. Together, it appears that language and communication deficits in ASD involve early and persisting deviant patterns of temporal cortical connectivity with other cortical regions. These early and persisting differences may help explain why treatment of core ASD symptoms is a challenge at all ages. The absence of neurotypical connectivity–behavior correlations in language- and social-related regions coupled with the presence of the highly atypical engagement of temporal–cuneus and temporal-precuneus connectivity may serve as biomarkers of early language and social deficits in ASD. Novel treatment approaches may be necessary to remodel and overcome these initial early-age, strikingly unusual neural networks for social and communication in ASD [60].

## Supplementary Information

**Additional file 1.** Supplementary Methods, Tables and Figure.

## Data Availability

The tidy data used in this study and R code for completing the analyses reported in this article are available at https://github.com/Yaqiongxiao/asdlanguage_rsfMRI.
