# Supplementary material for: Atypical functional connectivity of temporal cortex with precuneus and visual regions may be an early-age signature of ASD
Source: Mol Autism. 2023 Mar 10;14:11. doi: 10.1186/s13229-023-00543-8 (PMC10007788; doi:10.1186/s13229-023-00543-8)
Supplement: Supplementary file 1 — Additional file 1. Supplementary Methods, Tables and Figure. [file 13229_2023_543_MOESM1_ESM.pdf]

Supplementary Information

Methods

GeoPref eye tracking test

Eye tracking was conducted using Tobii software (Tobii Studio and Tobii Pro Lab), and fixation data were collected using a velocity threshold of 0.42 pixels/ms (Tobii Studio Tobii Fixation Filter) or 0.03 degrees/ms (Tobii Pro Lab Tobii IV-T Fixation Filter). Percent fixation to dynamic social images was used as an index of social visual attention and was computed by dividing fixation duration within an area of interest drawn around the dynamic social images by the total fixation duration across the entire video. In order to control for spatial biases, spatial location of stimuli presentation (left/right) was randomly assigned across subjects. A total of 28 different geometric/social scenes of variable duration (0.8–3.7 seconds) were presented and lasted 62 seconds.

Of the total 86 subjects with resting-state fMRI data, 65 (32 ASD/33 non-ASD) had moderate or good eye tracking performance and total looking time > 50%. The other 21 subjects either failed to complete the eye tracking session, or the data quality was poor. For the demographic characteristics and clinical testing scores in ASD and non-ASD subjects with and without successful eye tracking data, see **Table S2**. Fifty-nine of these 65 subjects completed the GeoPref test prior to the fMRI scan, and 6 completed the test after the fMRI scan.

ME-ICA procedure

Multi-echo principle component analysis (ME-PCA) was then applied as a dimensionality reduction technique before application of ME-ICA denoising. ME-ICA takes the ME-PCA reduced data and identifies independent components (ICs) that are then scored by pseudo-F statistics rho (ρ) and kappa (κ), which denote degree of non-BOLD and BOLD-related signal weightings based on TE-dependence analysis [2,3]. ICs with high rho (ρ) and low kappa (κ) are components of non-BOLD related signal and are removed as part of the denoising process, while ICs with low rho (ρ) and high kappa (κ) scores are components with high levels of BOLD-related signal and are retained. This ME-ICA denoising procedure has been shown to be effective at

substantially increasing temporal signal-to-noise ratio (tSNR) and successfully removes a large proportion of the head-motion related and other complex non-BOLD artifacts [2–6] and vastly improves test-retest reliability of functional connectivity measures [7].

Group comparisons of head motion

Head motion was quantified via framewise displacement (FD) [8]. The group average FD was minimal (mean FD < 0.11 mm) in both ASD (mean ± SD = 0.081 ± 0.036 mm, range 0.031–0.19 mm) and non-ASD subjects (mean ± SD = 0.11 ± 0.1 mm, range 0.038–0.58 mm). There were no significant group differences between ASD and non-ASD subjects ($t(84) = -1.57$, $p = 0.13$) in two-tailed two-sample *t*-tests.

Characteristics of Mullen and Vineland subtests

Mullen expressive and receptive language tap different aspects of language abilities, with expressive language tapping speaking and receptive language tapping auditory comprehension [9]. As expressive language is related to social skills [10,11], Vineland communication which taps language abilities including both expressive and receptive language and written language is an interaction of language and social constructs [12]. Vineland socialization indexes social functioning within age-normed contexts.

Calculation of Mullen adjusted age equivalent scores

For Mullen T scores, 23.5% of young children with ASD (12 out of 51) in our study performed at levels that were below 20, and in order to enhance accuracy for brain-behavior correlations, we used the age equivalent adjusted language scores to reflect their ability, rather than artificially assigning all such children a score of 20. Specifically, we converted all young children's raw scores to Mullen age equivalent values in months and then divided that by the child's chronological age at testing and multiplying by 100. Thus, a 20 month old child with a Mullen age equivalent of 20 months would have a score of 100, while a 40 month old child with the same Mullen age equivalent of 20 months would have a score of 50.

Table S1. Clusters showing significant connectivity–behavior relationships.

Group/Contrast	Behavior (scores)	Region	Peak MNI coordinates			Cluster size (voxels)
			x	y	z	
**Left temporal ROI**						
ASD subjects	Vineland communication	Left cuneus	-15	-67	3	155
**Right temporal ROI**						
Non-ASD vs. ASD subjects	Mullen age equivalent adjusted expressive language	ACC, DLPFC	8	40	16	211
		Right LPC	35	-60	36	95
Non-ASD vs. ASD subjects	Vineland communication	Right LPC	32	-60	36	142
Non-ASD vs. ASD subjects	Vineland socialization	Right LPC	35	-60	36	287
		Right cerebellum	22	-50	-38	242
		ACC, DLPFC	8	40	19	197
Non-ASD subjects	Mullen age equivalent adjusted expressive language	ACC, DLPFC	8	40	16	698
		Right LPC	25	-57	46	111
Non-ASD subjects	Vineland communication	ACC, DLPFC	8	40	16	145
		Right cerebellum	18	-50	-38	113
		Right LPC	32	-60	40	79
Non-ASD subjects	Vineland socialization	ACC, DLPFC	8	40	-16	584
		Right LPC	32	-60	40	234
		Right cerebellum	22	-47	-38	225
ASD subjects	Mullen age equivalent adjusted expressive language	Left precuneus	-5	-54	30	78

Note: Clusters were corrected for multiple comparisons with voxel-wise $p = 0.001$ and cluster size > 63 voxels (cluster-wise $p < 0.05$, FWE corrected). Abbreviations: ACC, anterior cingulate cortex; DLPFC, dorsolateral prefrontal cortex; LPC, lateral parietal cortex.

Table S2. Demographic characteristics and clinical testing scores in ASD and non-ASD subjects with and without successful eye tracking (ET) test.

	With ET test		Without ET test		With vs. without ET test		Cohen's *d*
	Mean (SD)	Range	Mean (SD)	Range	*t* value	*p* value	
**ASD subjects**							
**Sample size (M/F)**	25/7		16/3		$\chi2$ = 0.03	0.87	
**Age at MRI scan, months**	28.94 (9.17)	14–55	27.89 (9.46)	16–52	0.385	0.703	0.112
**Age at clinical tests, months**	27.62 (8.12)	12–45	27.33 (9.63)	15–51	0.111	0.912	0.034
**ADOS social affect**	14.12 (3.55)	6–20	13.47 (3.79)	8–19	0.607	0.548	0.179
**ADOS restricted and repetitive behavior**	5.38 (2.01)	2–9	5.84 (1.8)	3–8	-0.856	0.397	0.241
**ADOS Total**	19.5 (4.79)	8–27	19.32 (4.88)	11–27	0.131	0.896	0.038
**Mullen visual reception**	37.62 (13.41)	10–63	34.74 (13.9)	1–58	0.727	0.472	0.213
**Mullen fine motor**	37.81 (13.19)	20–60	38.74 (12.07)	20–57	-0.255	0.8	0.072
**Mullen receptive language**	33.25 (14.37)	11–59	25.47 (13.99)	1–56	1.9	0.065	0.546
**Mullen expressive language**	33.91 (16.61)	7–63	23.89 (14.82)	1–56	2.229	0.031	0.627
**Mullen early learning composite**	75.59 (19.9)	42–115	65.37 (20.03)	29–105	1.767	0.085	0.513
**Vineland communication**	82.81 (19.24)	35–126	79.42 (11.77)	55–99	0.781	0.439	0.201
**Vineland daily living skills**	87.44 (13.02)	63–116	84.68 (9.51)	68–100	0.868	0.39	0.232
**Vineland socialization**	85.88 (11.94)	57–108	77.79 (10.98)	60–97	2.461	0.018	0.697
**Vineland fine motor**	94.84 (10.05)	74–117	85.78 (8.22)	74–105	3.448	0.001	0.96
**Vineland adaptive behavior composite**	83.84 (12.69)	58–111	78.58 (7.79)	67–94	1.835	0.073	0.472
**Non-ASD subjects**							
**Sample size (M/F)**	21/12		0/2		$\chi2$ = 1.08	0.3	
**Age at MRI scan, months**	25.12 (8.03)	14–46	38.5 (7.78)	33–44	-2.357	0.231	1.667
**Age at clinical tests, months**	26.21 (7.84)	13–37	28.5 (17.68)	16–41	-0.182	0.885	-0.275
**ADOS social affect**	3.06 (1.64)	0–8	1.5 (0.71)	1–2	2.711	0.13	0.965
**ADOS restricted and repetitive behavior**	1.61 (1.52)	0–5	0.5 (0.71)	0–1	1.955	0.217	0.737
**ADOS Total**	4.67 (2.15)	0–9	2 (1.41)	1–3	2.498	0.194	1.254
**Mullen visual reception**	54.3 (12.11)	30–80	47.5 (0.71)	47–48	3.14	0.004	0.57
**Mullen fine motor**	49.85 (8.34)	35–67	50 (9.9)	43–57	-0.021	0.986	0.018
**Mullen receptive language**	47.24 (11.84)	23–73	37 (2.83)	35–39	3.566	0.022	0.878
**Mullen expressive language**	44.09 (12.14)	25–70	27 (5.66)	23–31	3.778	0.086	1.425
**Mullen early learning composite**	98.03 (16.57)	71–130	81.5 (7.78)	76–87	2.662	0.145	1.009
**Vineland communication**	96.67 (10.69)	70–122	89.5 (10.61)	82–97	0.927	0.51	0.67
**Vineland daily living skills**	96.58 (10.64)	76–122	91.5 (17.68)	79–104	0.402	0.755	0.465
**Vineland socialization**	96.67 (9.9)	79–126	95.5 (12.02)	87–104	0.135	0.914	0.117
**Vineland fine motor**	98.25 (9.8)	71–115	101 (1.41)	100–102	-1.374	0.194	0.285
**Vineland adaptive behavior composite**	95.18 (9.58)	79–128	91 (15.56)	80–102	0.376	0.769	0.426

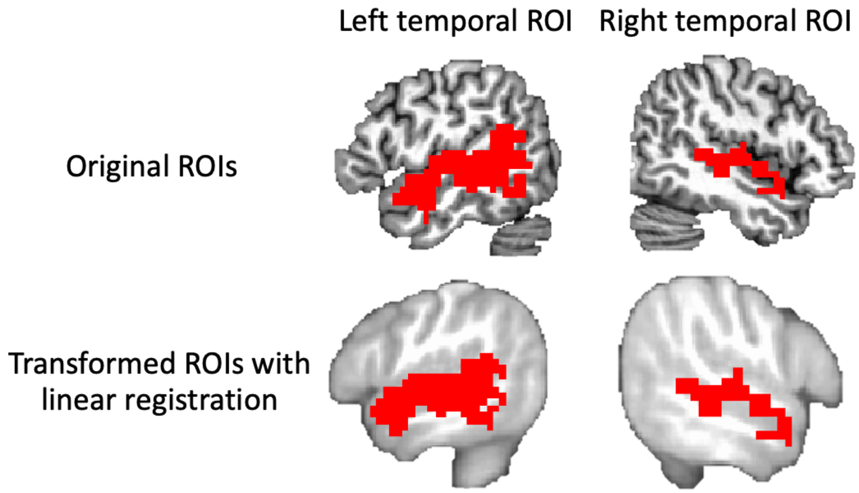

Figure S1. Regions of interest (ROIs) for the functional connectivity analysis. The upper row shows the original ROIs mapped onto the adult brain, and the lower row shows the transformed ROIs with linear registration mapped onto the 2-year-old toddler brain. The ROIs, i.e., left and right temporal regions, were extracted from the Neurosynth 'language' meta-analysis map (https://neurosynth.org/).

References:

1. Pierce K, Marinero S, Hazin R, McKenna B, Barnes CC, Malige A. Eye tracking reveals abnormal visual preference for geometric images as an early biomarker of an autism spectrum disorder subtype associated with increased symptom severity. Biol Psychiatry. 2016;79:657–666.

2. Kundu P, Inati SJ, Evans JW, Luh WM, Bandettini PA. Differentiating BOLD and non-BOLD signals in fMRI time series using multi-echo EPI. Neuroimage. 2012;60:1759–1770.

3. Kundu P, Voon V, Balchandani P, Lombardo M V., Poser BA, Bandettini PA. Multi-echo fMRI: A review of applications in fMRI denoising and analysis of BOLD signals. Neuroimage. 2017;154:59–80.

4. Kundu P, Brenowitz ND, Voon V, Worbe Y, Vertes PE, Inati SJ, et al. Integrated strategy for improving functional connectivity mapping using multiecho fMRI. Proc Natl Acad Sci U S A. 2013;110:16187–16192.

5. Lombardo M V., Auyeung B, Holt RJ, Waldman J, Ruigrok ANV, Mooney N, et al. Improving effect size estimation and statistical power with multi-echo fMRI and its impact on understanding the neural systems supporting mentalizing. Neuroimage. 2016;142:55–66.

6. Power JD, Plitt M, Gotts SJ, Kundu P, Voon V, Bandettini PA, et al. Ridding fMRI data of motion-related influences: Removal of signals with distinct spatial and physical bases in multiecho data. Proc Natl Acad Sci U S A. 2018;115:E2105–E2114.

7. Lynch CJ, Power JD, Scult MA, Dubin M, Gunning FM, Liston C. Rapid Precision Functional Mapping of Individuals Using Multi-Echo fMRI. Cell Rep. 2020;33:108540.

8. Power JD, Barnes KA, Snyder AZ, Schlaggar BL, Petersen SE. Spurious but systematic correlations in functional connectivity MRI networks arise from subject motion. Neuroimage. 2012;59:2142–2154.

9. Mullen EM. Mullen Scales of Early Learning. Circle Pine, MN, USA: American Guidance Service; 1995.

10. Schietecatte I, Roeyers H, Warreyn P. Exploring the nature of joint attention impairments in young children with autism spectrum disorder: associated social and cognitive skills. J Autism Dev Disord. 2012;42:1–12.

11. Pickard KE, Ingersoll BR. Brief Report: High and Low Level Initiations of Joint Attention, and Response to Joint Attention: Differential Relationships with Language and Imitation. J Autism Dev Disord. 2014;45:262–268.

12. Sparrow, S., Cicchetti, D. & Balla D. Vineland-II Scales Of Adaptive Behavior: Survey Form Manual. Circle Pines, MN, USA: American Guidance Service; 2005.
